# A simplified version of rapid susceptibility testing of bacteria and yeasts using optical nanomotion detection

**DOI:** 10.3389/fmicb.2024.1328923

**Published:** 2024-03-07

**Authors:** Maria I. Villalba, Vojislav Gligorovski, Sahand J. Rahi, Ronnie G. Willaert, Sandor Kasas

**Affiliations:** ^1^Laboratory of Biological Electron Microscopy (LBEM), Ecole Polytechnique Fédérale de Lausanne (EPFL), Université de Lausanne, Lausanne, Switzerland; ^2^International Joint Research Group VUB-EPFL BioNanotechnology & NanoMedicine (NANO), Brussels, Switzerland; ^3^Laboratory of the Physics of Biological Systems (LPBS), Institute of Physics, École Polytechnique Fédérale de Lausanne (EPFL), Lausanne, Switzerland; ^4^Research Group Structural Biology Brussels, Alliance Research Group VUB-UGhent NanoMicrobiology (NAMI), Vrije Universiteit Brussel (VUB), Brussels, Belgium; ^5^Centre Universitaire Romand de Médecine Légale (UFAM), Université de Lausanne, Lausanne, Switzerland

**Keywords:** nanomotion, optical microscope, bacteria, yeast, antimicrobials

## Abstract

We present a novel optical nanomotion-based rapid antibiotic and antifungal susceptibility test. The technique consisted of studying the effects of antibiotics or antifungals on the nanometric scale displacements of bacteria or yeasts to assess their sensitivity or resistance to drugs. The technique relies on a traditional optical microscope, a video camera, and custom-made image analysis software. It provides reliable results in a time frame of 2–4 h and can be applied to motile, non-motile, fast, and slowly growing microorganisms. Due to its extreme simplicity and low cost, the technique can be easily implemented in laboratories and medical centers in developing countries.

## 1 Introduction

In recent years, cellular nanomotion detection has proven to be an efficient way to rapidly determine bacterial sensitivity to antibiotics (Syal et al., 2017; Behera et al., 2019; Al-Madani et al., 2022; Rosłoń et al., 2022, 2023). The very first implementation of the method relied on atomic force microscopes (AFMs). Bacteria attached to an AFM cantilever were observed to induce nanometric scale oscillations of the lever as long as the organisms were alive, and these oscillations, referred to herein as nanomotion, stopped as soon as the organism died. Nanomotion was quickly applied to carry out rapid antibiotic susceptibility testing (AST) (Longo et al., 2013; Bennett et al., 2020; Caruana et al., 2022; Vocat et al., 2023). Such tests consist of exposing cantilever-attached organisms to various chemicals and assessing the organism’s viability by monitoring the cantilever oscillations. The method does not rely on the replication rate of the cells, allowing for AST results to be obtained in a time frame of 2–4 h (Stupar et al., 2017). It is important to note that this technique is equally effective with motile, non-motile, Gram-positive, Gram-negative, rapid, and slow-growing bacteria (Venturelli et al., 2021). The method was also demonstrated to be efficient in determining the rapid sensitivity of yeast and cancer cells to antifungals and antimitotic drugs, respectively (Lissandrello et al., 2014; Kasas et al., 2015; Wu et al., 2016; Stupar et al., 2021). Unfortunately, AFMs are relatively expensive devices and quite difficult to operate by inexperienced users. These two parameters seriously limit the applicability of the technique on a larger scale. In order to overcome these limitations, we recently demonstrated that simple optical microscopes can achieve nanomotion detection by using sophisticated image processing algorithms that monitor fungal or bacterial displacements with a sub-pixel resolution (Willaert et al., 2020; Villalba et al., 2023). In this case, single bacterium or yeast cell displacements are tracked using optical microscopy in a timeframe of approximately 10 s as a function of time after exposure to chemicals. The modification of the displacement pattern before and after the action of the drug permits assessing its effect on the viability or metabolism of the cells. The technique, referred to as optical nanomotion detection (ONMD), presents several advantages as compared to the traditional AFM-based method. Since it does not require the attachment of the cells to a cantilever and is a single-cell technique, it is much simpler to implement and significantly cheaper than its AFM-based ancestor. ONMD was shown to be an efficient and rapid AST method that can be applied to motile, non-motile, Gram-positive, Gram-negative, rapid, and slow-growing bacteria (Villalba et al., 2023) and yeast cells (such as Candida species) (Willaert et al., 2020; Radonicic et al., 2022, 2023).

In this study, we further improved and simplified the previously described ONMD method by replacing the previously used tracking algorithm (Willaert et al., 2020), which was based on cross-correlation, with a newly developed one. In this new approach, the displacement of cells in a bacterial and yeast population (100–500 cells) is recorded on a 10-s video under exposure to the antibiotic or antifungal drug. The drug’s effect on the organisms is assessed by highlighting the global population displacements between two consecutive frames. This methodology was briefly mentioned in a previous study (Villalba et al., 2023) but was never optimized or systematically tested with different types of organisms. In this study, we optimized the technique and applied it to motile, non-motile, Gram-positive, Gram-negative, rapid and slow-growing bacteria, and two different yeast species. We also addressed resistant and sensitive bacterial strains to a given antimicrobial. In all the previously cited cases, the technique revealed the sensitivity or resistance of the organisms in a very short time. It should also be emphasized that the method only requires a basic optical microscope with a camera and a laptop. The sample preparation is also very simple and does not require any specific equipment. We are convinced that due to its extreme simplicity, this variant of the ONMD method could be rapidly implemented in medical centers in developing countries.

## 2 Materials and methods

### 2.1 Bacteria and yeast cultivation

The bacteria *Staphylococcus aureus*, *Escherichia coli* (DH5α), and *Mycobacterium smegmatis* (mcc 155) were used in this study. *M. smegmatis* was cultured for 3 days on Luria–Bertani (LB) (Invitrogen™ 12,780,052) agar plates at 37°C. A 3-ml culture of LB broth with 0.1% v/v Tween 20 (Sigma-Aldrich, P1379) was used to grow the cells for 20 h at 160 revolutions per min (rpm) and 37°C. Cell suspension was washed with phosphate-buffered saline (PBS). After the wash step, the cells were concentrated by centrifugation at 3600 rpm for 5 min and resuspended in LB + 0.1% v/v Tween 20 (final OD_595_:0.75). The *E. coli* and *S. aureus* strains were grown for 24 h on LB agar at 37°C. An overnight subculture of colonies was performed in LB broth at 37°C and 160 rpm. The liquid cultures were centrifuged at 6000 rpm for 5 min and washed with PBS. To conduct the experiment, the suspension of cells was diluted in the fresh medium (OD_595_: 0.75).

The yeasts *Candida albicans* SC5314 and *Saccharomyces cerevisiae* W303 strains were cultured on yeast extract–peptone–dextrose (YPD) agar plates for 24 h at 30°C. YPD consists of 40 g/L of D-glucose (Gibco™ 15,023,021), 10 g/L of peptone (Millipore 82,303), 10 g/L of yeast extract, and 15 g/L of agar (plates only) diluted in distilled water. Colonies were re-cultured in 4 mL of YPD liquid medium and incubated overnight at 30°C and 160 rpm. An aliquot of the liquid culture was centrifuged at 3500 rpm for 5 min and diluted in fresh YPD broth. A 10-times dilution of the overnight cultures was prepared by centrifugation (3,500 rpm and 5 min) to perform the ONM measurements.

Half-maximal inhibitory concentrations (IC_50_) were determined using an optical density (OD) assay. *E. coli* and *C. albicans* were exposed overnight to ampicillin and fluconazole at different concentrations (Muñoz et al., 2017; Costa et al., 2020). The absorbance of the liquid cultures was measured at a wavelength of 600 nm. We defined the OD of the control culture (without antimicrobials) as 100% viable, and the “[inhibitor] vs. normalized response” analysis tool was used in GraphPad Prism 10 to calculate IC50 values and display the graphs.

### 2.2 Sensitivity test setup

**Figure 1:**
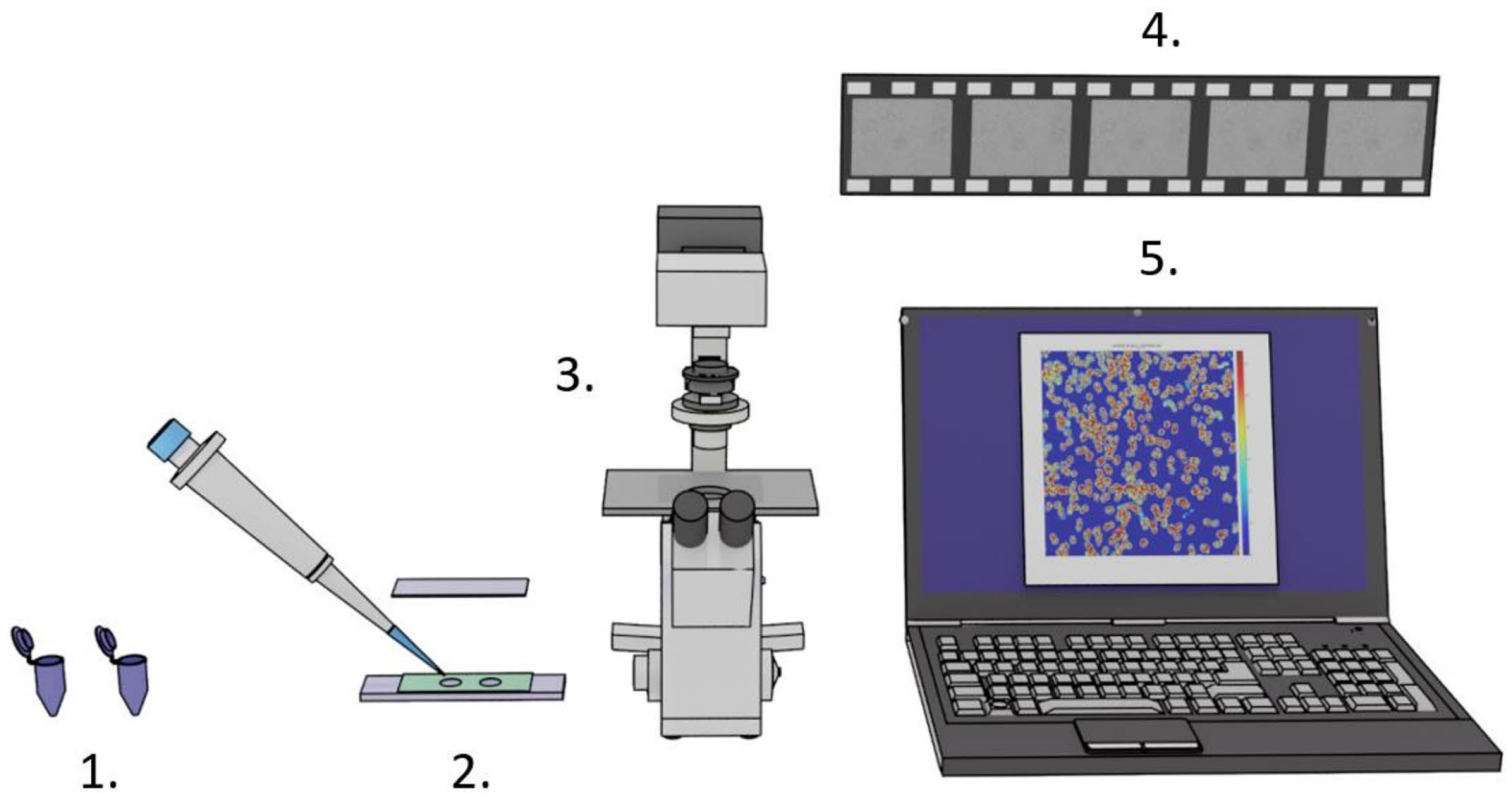
Setup for rapid ONMD measurements. 1. Control and antibiotic/antifungal exposed samples. 2. Analysis chambers composed of a sample holder, 5- or 10-μm-thick rubber tape (green), and a coverslip to close the analysis chambers. 3. An optical microscope equipped with a camera. 4. Several 10 s-long videos recording the organism’s displacements. 5. Computer with custom-made analysis software.

The required equipment is very basic, as depicted in Figure 1, and consists of two samples (i.e., a control and a drug-exposed sample), a custom-made analysis chamber, a camera mounted on an optical microscope, and a computer with ONMD analysis software.

### 2.3 Analysis chamber and sample preparation

The microfluidic device used in our experiments consists of a sample holder glass, a 5- or 10-μm-thick double-sided adhesive tape (Nitto, 5,600 and 5,601-L color: transparent), and a classical glass coverslip. The analysis chambers are manufactured by punching 3–5 mm holes in the adhesive tape with a desk paper punch. The rubber tape is stuck onto the sample holder glass, and 3 μL of the oil (HFE 7500 fLuorinated oil, 3MTM Novec™) is deposited in the wells and allowed to dry for 5 min. It resulted in a cell-repellent chamber surface, which prevented cells from adhering to it. A droplet of 0.65 μL–1.3 μL of the sample is deposited in the chambers (5- or 10-μm-thick chamber), which are finally sealed by sticking a coverslip atop them. Limiting the analysis chamber height to 5 and 10 μm permits keeping most of the organisms in the focal plane of the microscope.

### 2.4 Treatment and ONMD

A typical ONMD susceptibility measurement consisted of cultivating the organism of interest in test tubes in the presence (exposed sample) and absence of an antibiotic/antifungal drug (control sample). For each condition, aliquots (500 μl) of the cell suspension were incubated in 1.5 mL tubes (Eppendorf) during the treatment exposure time and the control sample. In particular, *S. aureus* (OD595:1) was incubated for 2 h at 37°C with or without vancomycin hydrochloride (CELLPURE, 0242.3) in dimethyl sulfoxide (DMSO; Sigma-Aldrich, D5879). *M. smegmatis* was exposed and not exposed (control) to 50 μg/mL of streptomycin (Sigma S6501) during a 5-h incubation at 37°C in a shaking condition (160 rpm). Using a cell suspension with an OD_595_:0.5 ratio, *E. coli* resistant and susceptible to ampicillin were exposed to 50 μg/mL of ampicillin (Sigma A0166, dissolved in water) for 3 h at 37°C shaking. In order to conduct yeast and antifungal treatment experiments, *C. albicans* was incubated with and without fluconazole (Sigma F8929) at a concentration of 100 μg/mL and *S. cerevisiae* with amphotericin B at a concentration of 250 μg/mL (Acros Organics 455,490,010). Both samples were incubated at 30°C for 5 h at 160 rpm.

After the treatment exposure to the chemical at a temperature of 30°C or 37°C (depending on the strain) in a shaker at 160 rpm, the samples of both the control and exposed samples are deposited in a microfluidic device consisting of two to several analysis chambers. The microfluidic device is inserted into a traditional optical microscope, and a video of the chambers is recorded for 10s. Eventually, the videos are analyzed using MATLAB (v. 2023a) software. The results are displayed as a bar chart and exported as a *txt* file.

### 2.5 Nanomotion video recording and data processing

**Figure 2:**
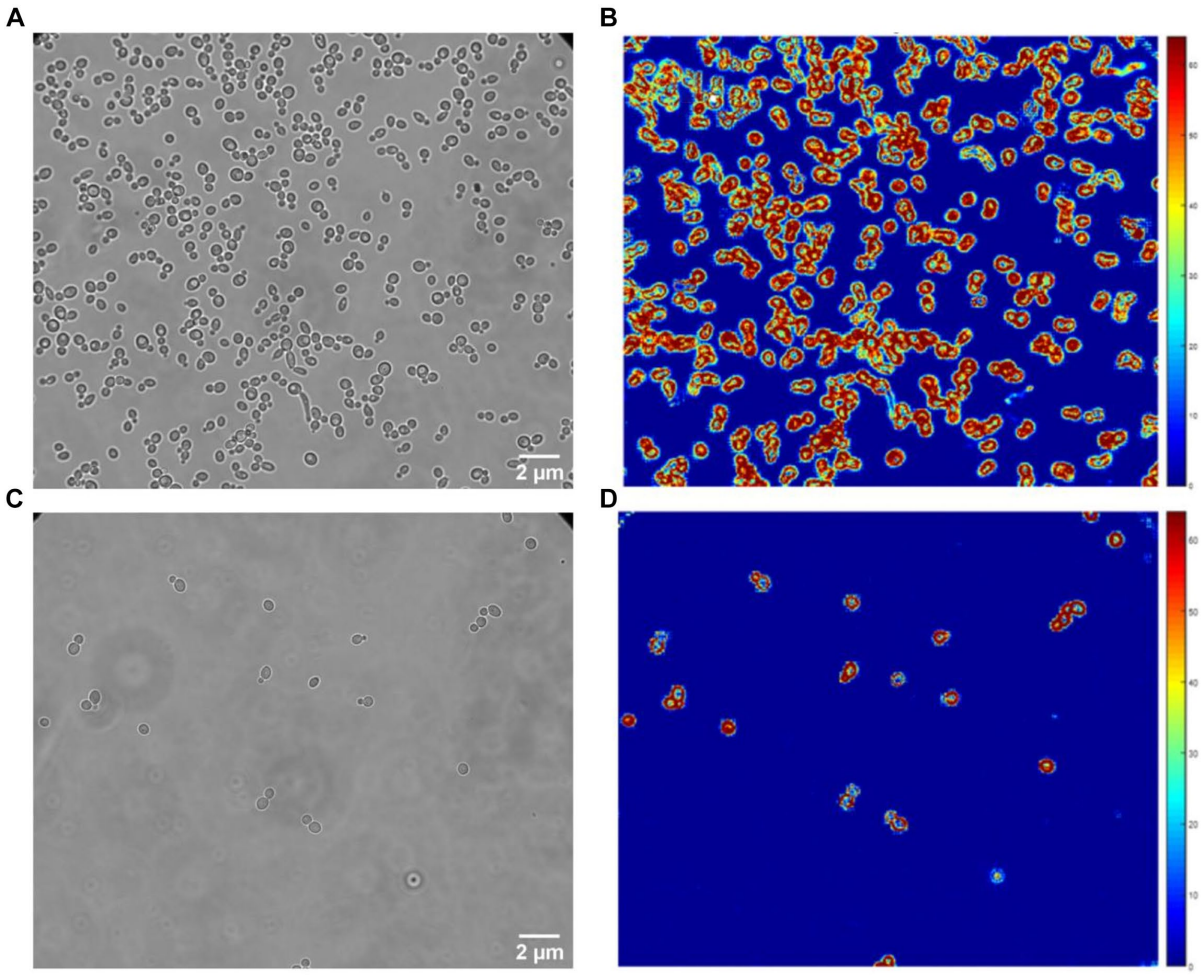
Classical optical **(A,C)** and Diff images **(B,D)** of *C. albicans*. Control **(A,B)** and fluconazole **(C,D)** exposed samples.

**Figure 3:**
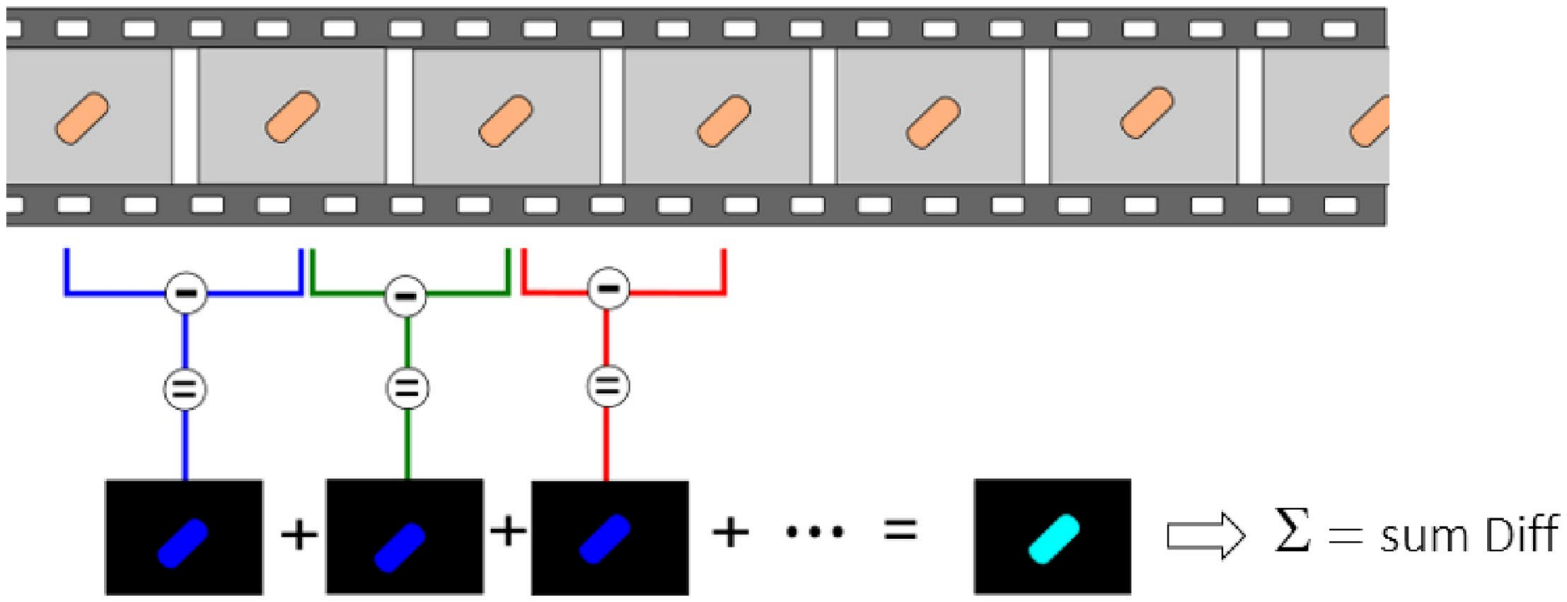
Ten s-long videos are recorded and processed to highlight minute pixel changes occurring between consecutive frames. Consecutive frames are subtracted from each other, and the sum of all these differences is made.

For these experiments, we used a Zeiss inverted traditional optical microscope (Observer Z.1) with a 63x oil immersion objective, coupled with a PCO Edge 5.5 camera. Several (3–5) 10 s-long videos of different fields of view were recorded at 30 fps of both the control and exposed samples for each replicate. Figure 2 depicts a typical field of view obtained with this equipment on *S. cerevisiae*. Figure 2A corresponds to the yeast imaged in its culture medium, and Figure 2C corresponds to the one exposed to fluconazole. One can clearly notice the difference in the number of cells present in the field of view. A MATLAB program processes the recorded videos and highlights variations in pixel intensity that occur between two consecutive frames, as depicted in Figure 3. The obtained image, referred to as the *Diff image,* highlights in false colors the pixels whose intensity varied during the recording. Figures 2B,D depict the *Diff Images* of Figures 2A,C. Eventually, the program calculates the sum of all the pixels composing the *Diff image* (i.e., *sum Diff*) on both exposed (*sum Diff Exposed*) and control samples (*sum Diff Control*). The sum of the pixels composing the Diff image corresponds to the optical nanomotion value expressed in arbitrary units. Comparing the ONMD value of the control with the exposed sample permits assessing bacterial/fungal sensitivity to the tested drug. Comparing these two numbers allows us to assess the bacterial /fungal sensitivity of the tested drug. We used the following rules for this purpose:

$\mathrm{Sum}\;\mathrm{Diff}\;\mathrm{control}=\langle \mathrm{Sum}\;\mathrm{Diff}\;\mathrm{exposed}-\rangle \mathrm{Resistant}$

SumDiffcontrol>>SumDiffexposed−>Sensitive

If several experiments are carried out in similar conditions, the results can be displayed as histograms, violin plots, or boxplots. On request, the image processing program is available for free.

## 3 Results

**Figure 4:**
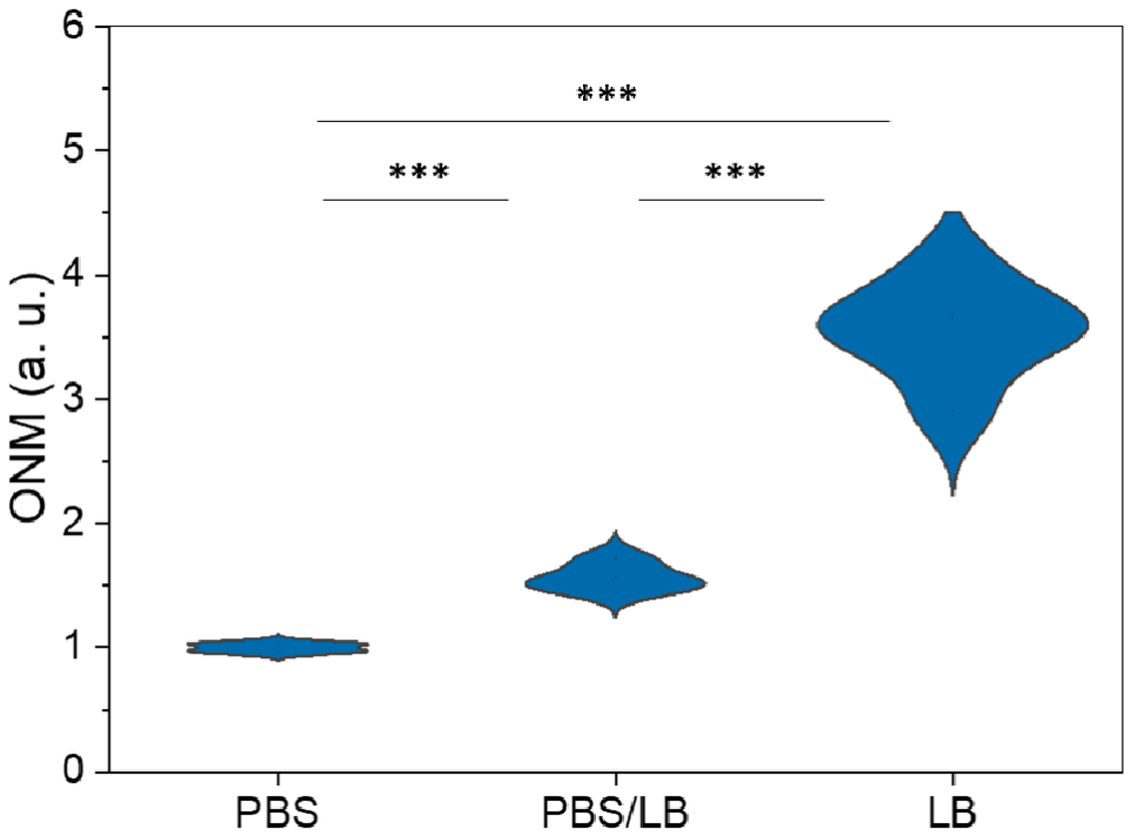
Optical nanomotion (ONM) of *E. coli* bacteria suspended in increasing nutrient concentration media. *** *p* < 0.001. PBS data consist of 4, PBS/LB in 4, and LB in a total of 6 videos.

The classical ONMD algorithm required the user to select individual cells to be tracked using a cross-correlation algorithm (Willaert et al., 2020). This methodology limits the number of cells that can be analyzed in a reasonable timeframe. In the new approach, the sum of the pixels composing the entire diff image is used to quantify nanomotion. Therefore, more microorganisms are considered, no cell recognition is needed, and the contribution of the user is limited to selecting videos to be processed. First, we tested the efficiency of the new technique to monitor the nanomotion of *E. coli* as a function of the nutrient concentration in the medium. The cells were exposed to 100% PBS, 50% PBS and 50% LB, and 100% LB. Figure 4 depicts the global nanomotion (sum Diff) of 4 (PBS and PBS/LB) and 6 (LB) videos and is displayed as violin plots. As observed, the nutrient concentration in the medium increases the sum Diff value, indicating that rapid ONMD (rONMD) also reflects *E. coli* metabolism since the largest nanomotion signal is recorded for the condition with sufficient nutrients (100% LB medium). These obtained results were comparable to those obtained using AFM-based nanomotion detection (Longo et al., 2013) and the “traditional” ONMD measurements (Willaert et al., 2020). In both these cases, a decrease in nanomotion was observed in bacteria exposed to nutrient deficiency conditions.

**Figure 5:**
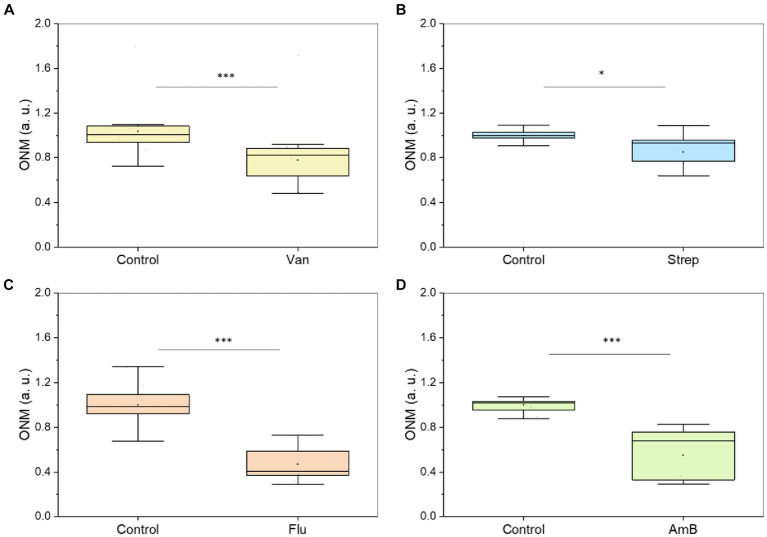
Different microorganisms exposed to an antibiotic or antifungal. **(A)** *S. aureus* – 50 μg/mL of vancomycin (2 h at 37 ᴼC) (five replicates), **(B)** *M. smegmatis* – 50 μg/mL of streptomycin (5 h at 37 ᴼC) (five replicates), **(C)** *C. albicans* 5,314–100 μg/mL of fluconazole (5 h) (five replicates), **(D)** *S. cerevisiae* – 200 μg/mL of amphotericin B (5 h) (five replicates). *p < 0.05, *** *p* < 0.001; Van: vancomycin, Strep: streptomycin, FLU: fluconazole, AmB: amphotericin B.

**Figure 6:**
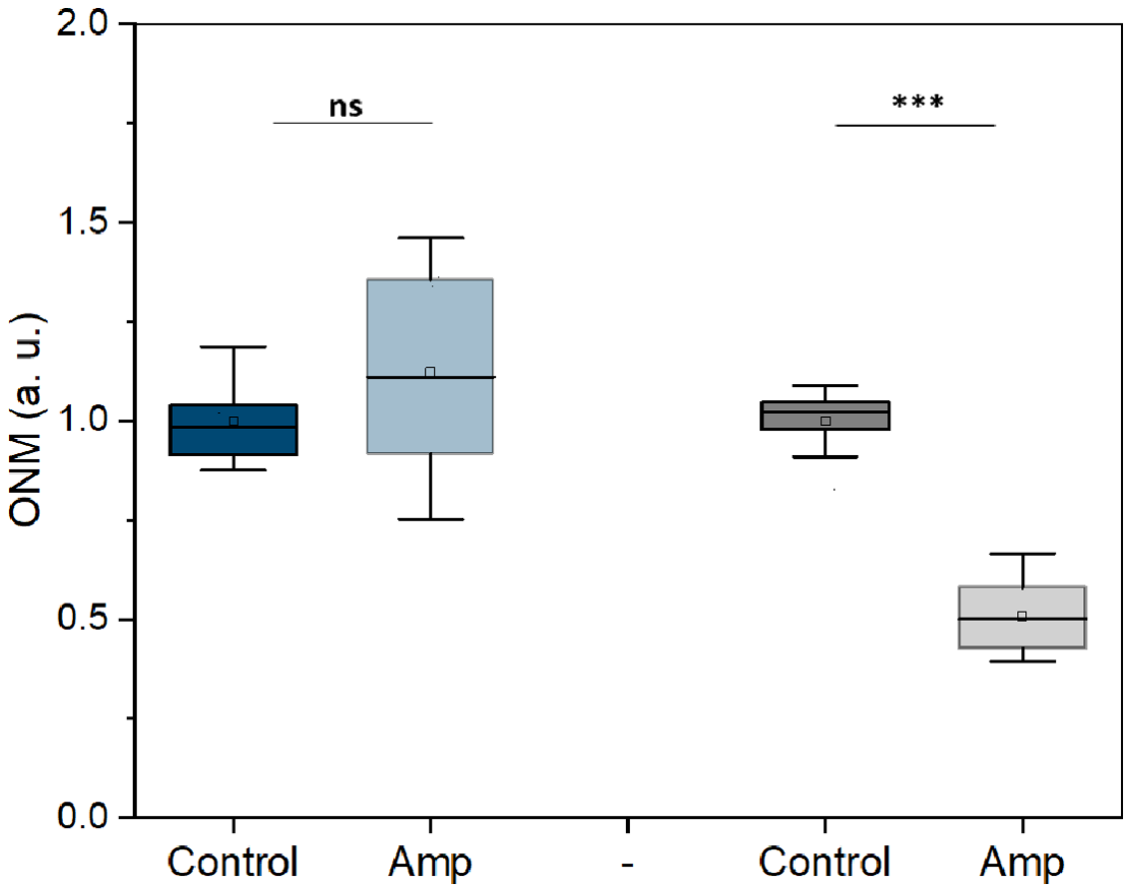
Nanomotion of *E. coli* resistant (blue) and sensitive (gray) to ampicillin after 3 h treatment (50 μg/mL at 37 ᴼC). ***p < 0.001, ns: no significant; Amp: ampicillin; three replicates.

Next, we used this rONMD technique to explore the effects of different antibiotics on motile (*E. coli*), non-motile (*S. aureus*), Gram-positive (*S. aureus*), Gram-negative (*E. coli*), and rapid (*E. coli* and *S. aureus*) and slow (*M. smegmatis*)-growing bacteria. We also compared resistant and sensitive strains (ampicillin effect on two different *E. coli* strains). In all cases, there was a significant difference in nanomotion between control and antibiotic-treated populations (Figures 5, 6), with a minimum difference value of 14.5% for *M. smegmatis* exposure to streptomycin and a maximum difference value of 49.38% for *E. coli* sensitive strain exposure to ampicillin (Figure 6). Additionally, the susceptibility of two yeast strains, i.e., *S. cerevisiae* and *C. albicans,* to the antifungals amphotericin B and fluconazole was also evaluated, where the difference between the ONM values of the control and antifungal-treated populations was 44.95 and 53.14%, respectively (Figure 5).

**Figure 7:**
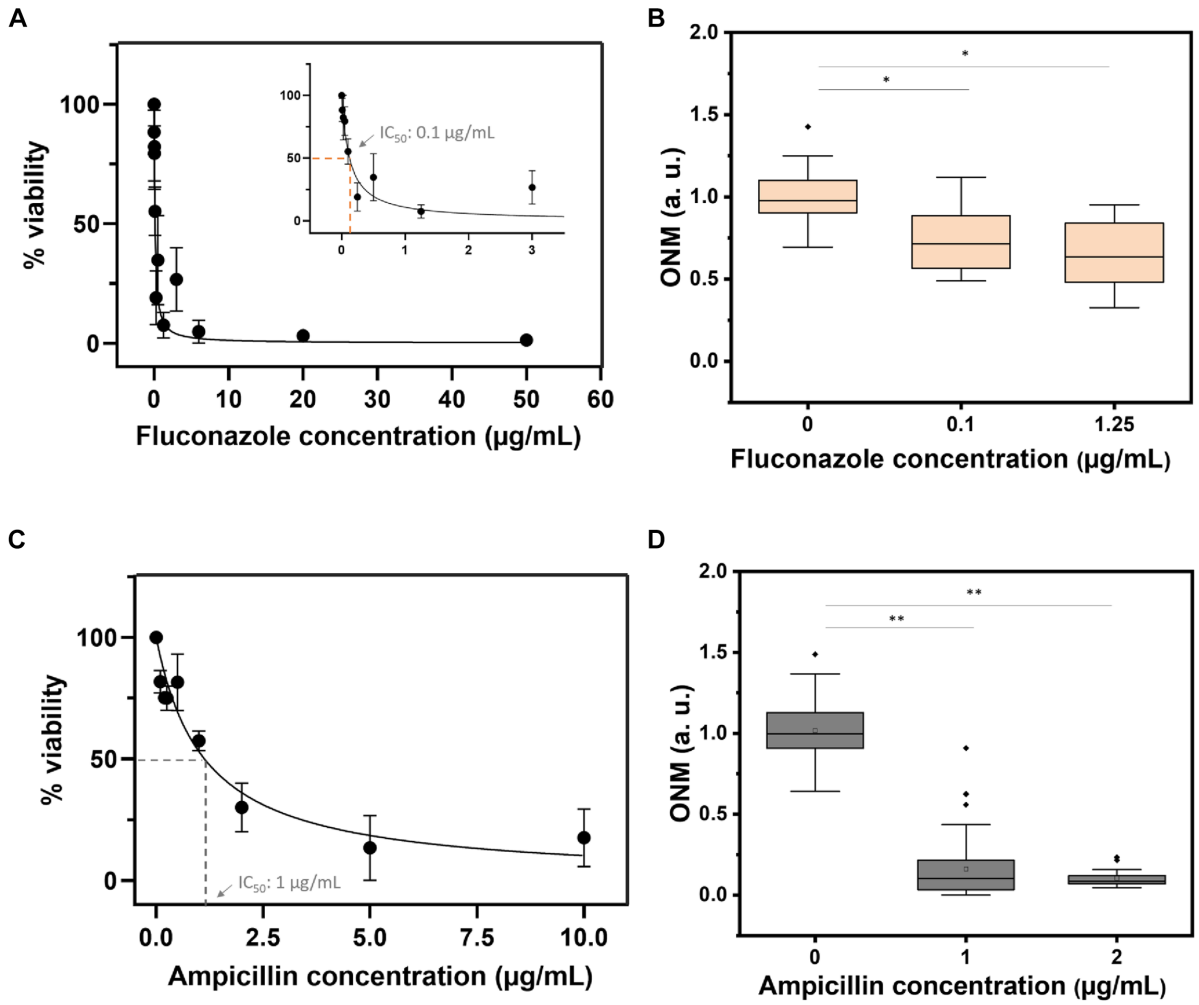
Viability curves based on the OD_600_ assay **(A,C)** and the rONMD boxplots **(B,D)** for *C. albicans* (upper panels) and *E. coli* (bottom panels) exposed to fluconazole and ampicillin, respectively. At least three independent experiments are represented by each data point. *p < 0.05, **p < 0.01.

Finally, we determined the IC_50_ of fluconazole and ampicillin using OD measurements of overnight cultures of *C. albicans* and *E. coli*. As depicted in Figure 7 the IC_50_ for *C. albicans* was 0.1 μg/mL (Zhao et al., 2013) and 1 μg/mL for *E. coli*. rONMD measurements of both microorganisms exposed to their respective IC_50_ presented statistically significant differences in their nanomotion as compared to the control cultures (not exposed to the drugs). These results further demonstrate the validity of rONMD measurements in assessing bacterial and yeast sensitivity to antimicrobial drugs.

## 4 Discussion

As demonstrated herewith, rONMD seems very comparable to the classical ONMD. It permitted monitoring of the vitality of microorganisms because there were differences between actively growing *E. coli* cells in a growth medium without nutrient limitations, cells with nutrient limitations (50/50 mixture of LB medium and PBS buffer), and cells in a medium with a lack of nutrients (in PBS buffer).

The rONMD method also allowed the detection of the sensitivity of the selected bacteria to antibiotics and *C. albicans* and *S. cerevisiae* cells to antifungals. The sensitivity testing consists of comparing a control sample with the sample exposed to the drug; therefore, it should be emphasized that two synergic phenomena contribute to increasing the sensitivity of the rONMD method. The first is the number of cells present in the field of view of the camera that will drop if the drug possesses a growth-inhibiting effect. The second is the well-described decrease in the nanomotion of the microorganisms following their exposure to the drug. The antibiotic/antifungal sensitivity test is very rapid, as resistant *E. coli* bacteria can be distinguished from sensitive ones in just 3 h (Figure 6). By facilitating the rapid detection of antimicrobial susceptibility, the method may prove to be extremely helpful in combating the evolution of antimicrobial resistance, one of the most pressing concerns in the modern world.

This new ONMD technique (rONMD) does not require monitoring isolated cell displacements, which considerably simplifies the data processing algorithm. It relies on comparing the displacement of cells in a large population exposed to antimicrobial drugs versus control samples using optical microscopy.

Importantly, the technique relies on very basic hardware and software and can be foreseen as easily implemented in medical centers of developing countries. The microfluidic device used as an analysis chamber can be manufactured on the spot in less than 5 min and does not require any specialized instrumentation (nor a clean room), except the 5-μm-thick double-faced rubber tape and a desk paper punch. Very basic low-cost optical microscopes can be used as imaging devices (Villalba et al., 2023), and if required, the imaging camera can be replaced by a mobile phone. Contrary to the previous ONMD sensitivity testing method, no sophisticated cell tracking computer software is required.

## Data availability statement

The raw data supporting the conclusions of this article will be made available by the authors, without undue reservation.

## Author contributions

MV: Conceptualization, Investigation, Methodology, Validation, Writing – review & editing. VG: Investigation, Writing – review & editing. SR: Funding acquisition, Resources, Writing – review & editing. RW: Conceptualization, Funding acquisition, Methodology, Resources, Validation, Writing – original draft, Writing – review & editing. SK: Conceptualization, Funding acquisition, Methodology, Project administration, Resources, Software, Supervision, Visualization, Writing – original draft, Writing – review & editing.
